# Peroxisomal Metabolism and Dynamics at the Crossroads Between Stimulus Perception and Fast Cell Responses to the Environment

**DOI:** 10.3389/fcell.2020.00505

**Published:** 2020-06-26

**Authors:** Luisa M. Sandalio, Maria Angeles Peláez-Vico, María C. Romero-Puertas

**Affiliations:** Department of Biochemistry and Molecular and Cellular Biology of Plants, Estación Experimental del Zaidín, Consejo Superior de Investigaciones Científicas (CSIC), Granada, Spain

**Keywords:** cross-talk, peroxisomes, posttranslational modifications, peroxules, pexophagy, reactive oxygen species, stress

Peroxisomes are highly dynamic, multifunctional plastic organelles whose metabolism, number and phenotype can change depending on developmental, environmental and metabolic requirements. However, the molecular mechanisms governing their plasticity and the role of these changes in development, and stress responses, some of the most challenging areas in the field of biology, are not fully understood. Since their discovery in 1959 by de Duve, information regarding new metabolic pathways associated with these organelles, has increased at an extraordinary rate (Pan et al., 2019). Plant peroxisomes are closely related to other organelles housing metabolic pathways, such as fatty acid β-oxidation, which provide energy during the initial stage of seedling growth by channeling fatty acids from oil bodies to peroxisomal β-oxidation, with acyl-CoA oxidase being one of the first enzymes involved in this process. Acetyl-CoA from fatty acid β-oxidation is then converted into C4 carboxilic acids by the glyoxylate cycle in seedlings. Fatty acid β-oxidation is also involved in important processes including synthesis of indoleacetic acid (IAA), jasmonic acid, ubiquinone, as well as secondary metabolites such as benzoic acid (BA) and phenylpropanoids (Pan et al., 2019). On the other hand, light triggers a shift in the peroxisomal metabolism and activates the photorespiration cycle, in which sugars are oxidized to CO_2_ in a complex pathway where physical contact between chloroplasts, peroxisomes and mitochondria is required. Other metabolic pathways in peroxisomes include ureide metabolism, polyamine and amino acid catabolism. However, peroxisomes are also an important source of ROS and NO which are associated with these metabolic pathways, in addition to the electron transport chain located in the peroxisomal membrane (Sandalio and Romero-Puertas, 2015). This explains why disturbances in any of these metabolic processes can trigger transitory changes in ROS and NO production which can regulate peroxisomal metabolism and also be perceived by the cell as an alarm, causing a specific rapid response (Sandalio and Romero-Puertas, 2015; Figure 1A). This response can be regulated at the post-translational level by NO- and ROS-dependent post-translational protein modifications, which is a very fast, efficient inexpensive strategy to regulate proteins and, therefore, metabolic pathways (Sandalio et al., 2019; Figure 1A). The transcriptional regulation of cell responses mediated by ROS-dependent peroxisomal sources has also been investigated; hundreds of genes, such as those involved in anthocyanin biosynthesis, pathogenesis, cell death and ubiquitin-dependent protein degradation, as well as those encoding kinases, including MAPKs, and heat shock proteins, have been identified as regulated by H_2_O_2_ produced during photorespiration (Queval et al., 2007; Chaouch et al., 2010; Sewelam et al., 2014; Su et al., 2018). However, no information is available on peroxisomal NO-dependent regulation of gene expression or on the protein/gene producing NO inside the organelle.

**Figure 1:**
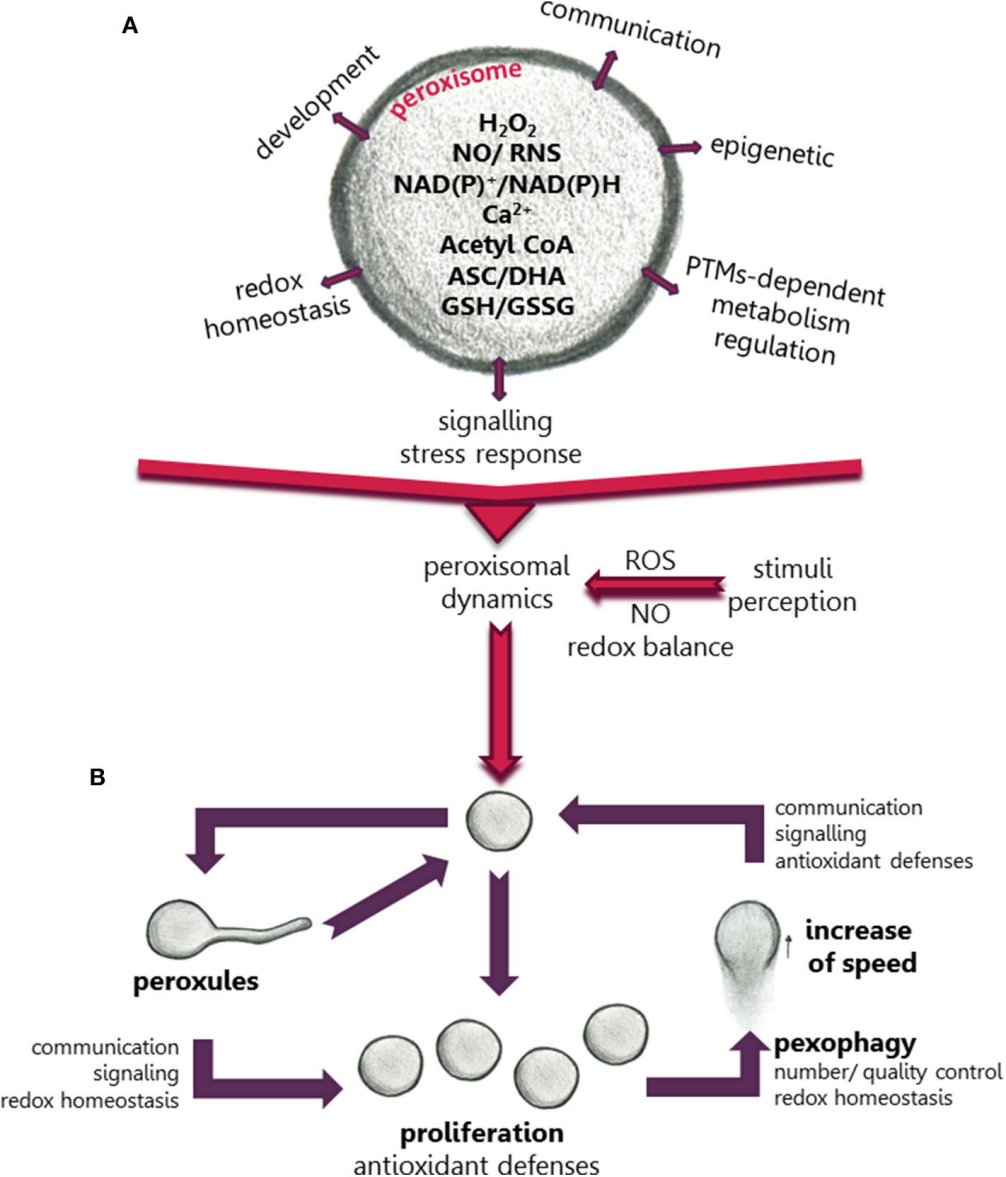
Scheme showing the role of peroxisomes in the regulation of metabolism, development and plant cell responses to environmental changes. **(A)** The regulatory role is mediated by ROS, NO, Ca^+2^, redox balance, and other compounds produced in peroxisomes. **(B)** Changes in peroxisomal dynamics and function in response to environmental stimuli mediated by changes in cellular ROS accumulation.

## Post-Translational Modifications Regulate the Peroxisomal Proteome and Its Metabolism

PTMs can be regarded as an interface between perception of changes in the environment and the rapid cellular responses to these changes. They can regulate protein activity, localization, degradation, and inter-protein interactions, giving rise to rapid finely-tuned regulation of protein functionality and, therefore, rapid changes in metabolic pathways and signaling processes (Hashiguchi and Komatsu, 2016). This regulation is especially important in plants subjected to changeable environments. Synergistic and antagonistic interplay between PTMs has led to a higher degree of complexity in the regulation of the perception of environmental changes and of specific plant cell responses. Recently, a meta-analysis of PTMs of the peroxisomal proteome (Sandalio et al., 2019) showed a high degree of possible PTM-dependent regulation of most metabolic pathways in peroxisomes, including phosphorylation, carbonylation, sulfenylation, persulfidation, *S*-nitrosylation, nitration, acetylation, and ubiquitination (Sandalio et al., 2019). This multilevel PTM regulation could explain the plasticity of peroxisomes and their capacity to rapidly respond to changes in their environment and to regulate metabolic pathways in organelles sharing metabolites with peroxisomes, such as mitochondria, lipid bodies and chloroplasts (Figure 1A). Peroxisomal PTMs can even act as on/off switches to channel metabolites to different metabolic pathways (Ortega-Galisteo et al., 2012; Romero-Puertas and Sandalio, 2016). Peroxisomal protein phosphorylation, and its role in regulating different peroxisomal events have been elegantly reviewed by Kataya et al. (2019). The potential peroxisomal targets for ROS- and NO-dependent PTMs have also been studied (Sandalio et al., 2019). However, although different peroxisomal proteins have been identified as potential targets of protein acetylation, which has been receiving much attention in recent years, its precise mechanisms and role played in peroxisomes are little understood (Drazic et al., 2016; Sandalio et al., 2019). Acetyl-CoA, an essential component in fatty acid β-oxidation commonly found in peroxisomes, could be involved in epigenetic modifications (Figure 1A). Thus, the Arabidopsis mutant deficient in acyl CoA oxidase 4 (*Atacx4)* shows a decrease in nuclear histone acetylation and an increase in DNA methylation, with similar results being obtained for Arabidopsis mutants deficient in the multifunctional protein MFP2 (*Atmfp2*) and ketoacyl-coenzyme A thiolase (*Atkat2*) (Wang et al., 2019). In mammalian cells, peroxisomal acetyl-CoA also plays a role in mitochondrial protein acetylation (Eisenberg-Bord and Schuldiner, 2017).

## Peroxisomal Dynamics Govern Fast Responses to Stress

Plant peroxisomes can change their size, morphology, number and even speed of movement (Figure 1B), although why, how and when these changes occur, as well as their advantages and disadvantages in terms of tolerance and acclimation are not well-understood. Peroxisomes move along actin filaments (Mathur et al., 2002) which requires myosin motor proteins (Jedd and Chua, 2002; Perico and Sparkes, 2018). PEROXISOME AND MITOCHONDRIAL DIVISION FACTOR1 (PMD1) acts as an actin-binding protein connecting the peroxisome to the cytoskeleton and is involved in peroxisomal division and the cellular distribution of peroxisomes under stress conditions. Although this connection appears to be regulated by the MPK17 kinase, experimental evidence is yet lacking that PMD1 is a target of MPK17 (Frick and Strader, 2018). In mammalian cells, peroxisomal movement is microtubule-dependent and is mediated by kinesin and dynein motors (Castro et al., 2018). The mitochondrial Rho GTPase 1 (MIRO1) has also been identified as an adaptor for peroxisome motor protein association which regulates the proliferation of peroxisomes and their motility (Castro et al., 2018). In Arabidopsis plants the motility of peroxisomes has also been correlated with the motility of ER (Barton et al., 2013).

Plant peroxisome abundance is governed by (1) biogenesis, associated with physiological processes and division (fission) of a preexisting peroxisome, (2) proliferation, which is related to stress responses, and (3) pexophagy, a selective degradation mechanism of peroxisomes by autophagy (Olmedilla and Sandalio, 2019). Proteins involved in peroxisome biogenesis and maintenance are called peroxins (PEXs) (Kao et al., 2018). Peroxisome proliferation involves peroxisome elongation, constriction and fission regulated by PEX11(a-e), GTPases called dynamin-related proteins (DRPs), and fission proteins (e.g., FIS1) (Rodríguez-Serrano et al., 2016; Pan et al., 2019). FIS1A and FIS1B are shared by peroxisomes and mitochondria, and DRP3 regulates peroxisomal and mitochondrial fission, while DRP5B is involved in the fission of peroxisomes and chloroplasts (Kao et al., 2018), indicating a highly coordinated regulation of organelle abundance.

Peroxisome proliferation has been observed in response to several abiotic stresses: high light (Desai and Hu, 2008), ozone (Oksanen et al., 2003), clofibrate (Nila et al., 2006; Castillo et al., 2008), salinity (Mitsuya et al., 2010), cadmium (Romero-Puertas et al., 1999; Rodríguez-Serrano et al., 2016) and drought (Ebeed et al., 2018); others factors such as the herbicide 2,4-dichlorophenoxyacetic acid (2,4-D) do not alter the number of peroxisomes (McCarthy et al., 2001), while jasmonic acid treatment reduces the number and increased their size (Castillo et al., 2008). Under these stress conditions, plant peroxisome proliferation can be considered a protective response probably to cope with ROS overflow in cell compartments thanks to major enzymatic and non-enzymatic antioxidant defenses in these organelles (Figure 1B). However, tobacco plants overexpressing the xenopus peroxisome proliferator-activated receptor α (α*PPAR*), regulating fatty acid β-oxidation and peroxisome proliferation, show constitutively larger peroxisome populations (Nila et al., 2006; Mitsuya et al., 2010), although this does not protect against salinity (Mitsuya et al., 2010) or clofibrate (Nila et al., 2006).

The role of changes in peroxisome dynamics under stress conditions can be elucidated by time course analysis of these changes. Sinclair et al. (2009) showed that, after a few minutes of treatment with H_2_O_2_, peroxisomes produce extensions or protrusions called peroxules, which progress over time with peroxisome elongation and further proliferation. Rodríguez-Serrano et al. (2016) demonstrated that peroxules were induced after 15 min of Cd treatment (Figure 1B) due to an increase in ROS production by NADPH oxidases, and peroxules formation was regulated by PEX11a. When producing peroxules, peroxisomes are immobile, suggesting that they are tethered to another organelle, with PEX11a representing a good candidate for mediating inter-organellar docking (Rodríguez-Serrano et al., 2016). The role of ROS in the formation of these structures could be related to changes in membrane elasticity (Sinclair et al., 2009), although PEX11a could also be regulated by redox-dependent PTMs that activate tethering and changes in the peroxisomal membrane. However, proteome analysis showed that PEX11a may be a target of phosphorylation rather than redox PTMs (Sandalio et al., 2019), while the possibility that PEX11a acts as a ROS sensor cannot be ruled out. Peroxules have been associated with peroxisome proliferation (Sinclair et al., 2009; Figure 1B), although Rodríguez-Serrano et al. (2016) suggested that they do not always cause proliferation and may be involved in regulating ROS accumulation and ROS-dependent signaling transduction (Rodríguez-Serrano et al., 2016; Figure 1B). Therefore, peroxules could be regarded as part of a strategy to increase the peroxisomal surface favoring metabolite exchange (such as H_2_O_2_, NO, Ca^2+^, acetyl CoA and lipids) with other organelles which would explain the physical connection observed with the ER, mitochondria and chloroplasts (Sinclair et al., 2009; Sandalio et al., 2013; Rodríguez-Serrano et al., 2016; Mathur et al., 2018). Peroxules are also involved in protein transport such as the transfer of the sugar-dependent 1 (SDP1) lipase from the peroxisomal membrane to the lipid body (Thazar-Poulot et al., 2015).

The components of the tethering complex between peroxisomes and other organelles have not been identified in plants. However, the peroxisomal Zn RING finger of PEX10 in Arabidopsis interacts with the outer membrane of the chloroplast envelope which is necessary for full photorespiration functionality (Schumann et al., 2007). The role of PTMs in the regulation of protein-protein interactions at inter-organellar contact site remains a fascinating and unexplored area of study. It is reasonable to assume that tethering is regulated by specific PTMs. For example, PEX10 is a candidate target for phosphorylation (Sandalio et al., 2019). Although peroxules have not been observed in mammalian cells under control conditions, a similar structure has been reported in PEX5-deficient fibroblasts expressing mitochondrial MIRO1 (Castro et al., 2018).

Following the time course of cell responses to Cd after peroxule formation, peroxisomes elongate, constrict and divide, reaching proliferation after 3 h of treatment with Cd (Rodríguez-Serrano et al., 2016). Longer treatment periods (24 h) considerably increase the peroxisomal speed of movement which is regulated by ROS produced by NADPH oxidases and Ca^2+^ ions (Rodríguez-Serrano et al., 2009; Figure 1B). This could improve antioxidant defenses in places where Cd induces ROS accumulation or favors signal transduction and metabolite exchange in different parts of the cell (Rodríguez-Serrano et al., 2009). Although information on the role of peroxisomal motility is scarce, peroxisomal movement has been reported to play an important role in myosin loss-of-function Arabidopsis mutants where the inhibition of organelle movement negatively affected plant growth (Ryan and Nebenführ, 2018).

Peroxisomal homeostasis is essential for cell viability, and excess, damaged and obsolete peroxisomes need to be degraded by selective autophagy (pexophagy). Pexophagy could be a solution for regulating basal levels of organelles and after stress-induced proliferation by mainly degrading oxidized peroxisomes (Yoshimoto et al., 2014; Calero-Muñoz et al., 2019; Figure 1B). Interestingly, the Arabidopsis mutants *Atatg2*, At*atg5, and* At*atg7*, which are deficient in autophagy, accumulate peroxisomes rather than other organelles such as mitochondria, thus confirming the importance of peroxisomal homeostasis (Yoshimoto et al., 2014; Calero-Muñoz et al., 2019; Figure 1B). Pexophagy also regulates root meristem development by regulating ROS and IAA homeostasis (Huang et al., 2019) and controls guard cell ROS homeostasis, facilitating stomatal opening (Yamauchi et al., 2019).

In summary, peroxisomes perceive changes in their environment, are involved in signaling and crosstalk between other organelles, ROS homeostasis regulation and influence cellular decision-making, involving nuclei, mitochondria and chloroplasts, through small molecules, such as H_2_O_2_, NO, and acetyl-CoA. In addition, the redox couples GSH/GSSG and ASC/DHA could also regulate signaling processes (Foyer and Noctor, 2011). Sequential changes in the morphology, number and velocity of these organelles contribute to these processes in a complex ROS- and probably NO-regulated manner.

## Author Contributions

LS: devised and wrote the article. MP-V and MR-P: contributed with some information. MP-V: drowed the figure. All authors contributed to the article and approved the submitted version.

## Conflict of Interest

The authors declare that the research was conducted in the absence of any commercial or financial relationships that could be construed as a potential conflict of interest.
